# A Psychometric Evaluation of the Intrapersonal Problems Rating Scale Among Older Adults

**DOI:** 10.1093/geroni/igaf122.3381

**Published:** 2025-12-31

**Authors:** Colleen Mock, Daniel Segal, Andrew Lac

**Affiliations:** University of Colorado Colorado Springs, Shaker Heights, Ohio, United States; University of Colorado Colorado Springs, Colorado Springs, Colorado, United States; University of Colorado Colorado Springs, Colorado Springs, Colorado, United States

## Abstract

**Introduction:**

Interpersonal difficulties have long been connected to worse psychological functioning among older adults. However, the understanding of intrapersonal problems in older adults is limited. A psychometric evaluation of intrapersonal problems and their relation to diverse psychological constructs among older adults is needed.

**Method:**

Older adults (N = 250; 50.4% women) completed the following self-report measures: Intrapersonal Problems Rating Scale, Levels of Personality Functioning Scale-Brief Form 2.0, Brief Geriatric Suicide Ideation Scale, Geriatric Anxiety Scale, Geriatric Depression Scale-Short Form, Depression Anxiety Stress Score-21, Michigan Alcohol Screening Test-Geriatric Version, Insomnia Severity Index, and the Webexec. The study aimed to further examine intrapersonal functioning and its relations to well established constructs of psychopathology and to provide an initial psychometric evaluation of the IPRS in older adults.

**Results:**

Measures of internal consistency were good to excellent for IPRS total and the 7 IPRS domain scores. Bivariate correlations revealed medium to large positive correlations between IPRS total scores with all other measures, ranging from .44 (IPRS with MAST-G) to .80 (IPRS with GAS). Gender differences were also found, with women reporting lower domain scores in externalizing, fantasy proneness, and apathy than men with small effect sizes across domains.

**Discussion:**

The internal reliability and convergent validity analyses of the IPRS provide solid psychometric evidence in support of the use of the IPRS as a measure of intrapersonal functioning among older adults. Findings also reveal that deficits in intrapersonal functioning are strongly associated with worse psychological functioning across several important psychopathology and cognitive domains.

